# 肺术后咳嗽评估—中文版莱斯特咳嗽量表的应用价值

**DOI:** 10.3779/j.issn.1009-3419.2017.06.04

**Published:** 2017-06-20

**Authors:** 志华 徐, 嵘嘉 林, 国卫 车, 明铭 王, 艳丽 戢, 鹏飞 李, 梅 杨

**Affiliations:** 610041 成都，四川大学华西医院胸外科 Department of Thoracic Surgery, West China Hospital, Sichuan University, Chengdu 610041, China

**Keywords:** 胸腔镜肺部手术, 中文版莱斯特咳嗽量表, 术后咳嗽, 肺部疾病, Video-assisted thoracoscopic surgery, The Mandarin Chinese version of the Leicester Cough Questionnaire, Cough, Pulmonary disease

## Abstract

**背景与目的:**

中文版莱斯特咳嗽量表（Mandarin Chinese version of the Leicester Cough Questionnaire, LCQ-MC）是评估咳嗽的主要方法，本研究探讨LCQ-MC能否用于客观评价肺部疾病患者术后咳嗽。

**方法:**

选取2015年9月-2016年4月间四川大学华西医院胸外科单个医疗组收治的例行胸腔镜肺部手术的患者并进行问卷调查，问卷分别于术前与术后填写。分析LCQ-MC值、朗巴赫α系数等统计学方法。

**结果:**

① LCQ-MC值在术前（19.57±1.73）显著高于术后（17.71±2.72）（*P*=0.041）。②克朗巴赫系数α系数在术前（0.87）和术后（0.89）均大于0.7。③术前LCQ-MC值在术后出现咳嗽组（19.31±1.84）显著低于术后无咳嗽组（19.97±1.46）（*P*=0.038）；术后LCQ-MC值在术后出现咳嗽组（16.67±2.91）显著低于术后无咳嗽患者（19.30±1.32）（*P*=0.001）。④肺叶切除术组患者术后LCQ-MC分值（17.75±2.51）和非肺叶切除术组患者（17.79±3.04）无明显统计学差异（*P*=0.936）。

**结论:**

肺疾病患者胸腔镜术后咳嗽情况可以应用LCQ-MC评估。

## Abstract (en)

**Background and objective:**

The Mandarin Chinese version of the Leicester Cough Questionnaire (LCQ-MC) is a symptom specific questionnaire designed to assess the impact of cough severity, a major symptom of postoperative patients undergoing lung resection is cough. The aim of this study is to validate the LCQ-MC in patients with lung operation.

**Methods:**

Totally 121 patients undergone the lung operation of single medical team, from September 2015 to April 2016 in the Thoracic Surgery Department of West China hospital Sichuan University, were investigated by LCQ-MC before and after operation. We analyzed and calculated the preoperative and postoperative scores of LCQ-MC and Cronbach α.

**Results:**

① The mean LCQ-MC score in preoperative (19.57±1.73) was significant higher than postoperative (17.71±2.72) (*P*=0.041). ②The Cronbach α in preoperative (0.87) and postoperative (0.89) was significant higher than 0.7. ③The preoperative scores of LCQ-MC (19.31±1.84) was significantly lower in postoperative cough group than in without postoperative cough group (19.97±1.46) (*P*=0.038). The postoperative scores of LCQ-MC (16.67±2.91) was significantly lower in postoperative cough group than in without postoperative cough group (19.30±1.32) (*P*=0.001). ④There was no statistical difference (*P*=0.936) between postoperative LCQ-MC score (17.75±2.51) in lobectomy group and non-lobectomy group (17.79±3.04).

**Conclusion:**

The LCQ-MC can assess the condition of cough after thoracoscopic surgery in patients with pulmonary disease.

肺部疾病患者术后及出院后生活质量的改善是加速康复外科的重要组成部分，而咳嗽是肺术后患者主要的临床症状^[1]^。客观评估肺术后患者的咳嗽程度不但可以指导术后正确的治疗，也有助于围手术期处理方法的改进（如术前肺康复及手术方法等）^[2]^。现有的研究表明咳嗽是机体的防御反射，有利于清除呼吸道分泌物和有害因子，但频繁剧烈的咳嗽对患者的工作、生活和社会活动造成严重影响^[3]^。中文版莱斯特咳嗽量表（Mandarin Chinese version of the Leicester Cough Questionnaire, LCQ-MC）用于评估肺部手术后患者的咳嗽情况已被证实能有效地评估急性咳嗽和慢性咳嗽患者的生活质量情况^[4, 5]^。然而，将LCQ-MC应用于肺疾病患者术后咳嗽的情况的研究尚未报道。本文通过LCQ-MC对121例肺部疾病患者手术前后咳嗽情况调查，探讨应用LCQ-MC评估肺部手术后患者的咳嗽情况的临床价值。

## 1 资料与方法

### 1.1 临床资料

选取2015年9月-2016年4月间四川大学华西医院胸外科单个医疗组收治的127例肺部疾病患者。纳入标准：①年龄18岁-78岁；②胸腔镜（video-assisted thoracoscopic surgery, VATS）肺叶、肺段或肺楔形肺部切除手术；③术后病理诊断明确；④术前签署知情同意书。排除标准：①术前2周内因咳嗽应用药物治疗者；②手术过程中转开胸、出血量大于1, 000 mL或二次手术；③全肺切除患者或不同意调查患者；④术前合并有空洞型结核、支气管扩张症及哮喘患者和明显咳嗽症状患者（VAS评分 > 60分）；⑤一秒用力呼气容积（forced expiratory volume in one second, FEV_1_）实测值小于1.0 L/S或FEV_1_/用力肺活量（forced vital capacity, FVC）小于60%。

排除6例，最终纳入121例患者，平均年龄为（56.22±10.35）岁。其中男性55例，平均年龄（55.71±9.17）岁；女性66例，平均年龄（56.65±11.30）岁。从不吸烟者76例，吸烟者45例，其中400年支以上（35例，28.93%），400年支以下（10例，8.26%）。肺癌患者79例，腺癌69例，鳞癌7例，腺鳞癌2例，大细胞癌1例；术后分期采用国际抗癌联盟（Union for International Cancer Control, UICC）2009，其中Ⅰ期+Ⅱ期73例，Ⅲ期+Ⅳ期6例。肺良性疾病患者42例，其中炎性假瘤17例，炎性肉芽肿7例，结核球9例，硬化性血管瘤6例，错构瘤3例。手术方式均采用胸腔镜手术：肺癌患者均行肺叶切除术或肺段切除+系统淋巴结清扫术，肺良性疾病患者行肺楔形切除或肺叶切除术。其中肺叶切除术73例，肺段切除18例，肺楔形切除30例。术后出现明显咳嗽症状患者为73例（60.33%, 73/121），未出现明显咳嗽症状患者48例（39.67%, 48/121）。

### 1.2 手术方法

VATS手术方式应用单向式胸腔镜肺叶切除法+系统淋巴结清扫^[6]^。系统淋巴结清扫左侧必须清扫第5、6、7、8、9、10组淋巴结，右侧包括第2、3、4、7、8、9、10组淋巴结^[7]^。若术中诊断为良性肿瘤则不清扫淋巴结。术后引流应用双根16F尿管，置入第3或4、7肋间镜孔^[8]^。

### 1.3 术后处理

拔管后均鼓励患者咳嗽，必要时刺激患者咳嗽。术后第2天均行胸部照片，若无漏气且每天引流量 < 300 mL，肺已复张则拔除引流管。术后疼痛处理应用镇痛泵（5 mg loading dose followed by 1.0 mg/h-1.5 mg/h），早期鼓励患者下床活动。必要时应用非甾体类止痛药（泰勒宁或芬必得）。镇痛泵在引流管拔除时也一并停止使用^[9]^。

### 1.4 咳嗽评价方法

LCQ-MC分为生理、心理和社会三个维度，一共19道题，每道题7个选项（正向计分，1-7个等级，分数越高表示咳嗽程度越轻）^[5]^。各维度的得分由各维度题目分值取平均值（1分-7分），总分为三个维度得分之和（3分-21分）。本研究中，121例患者均在2位经过培训的医务人员进行指导下，分别于手术前(术前)，手术后（术后）完成LCQ-MC。术后咳嗽应用视觉模拟评分法（visual analogue scale, VAS），当尺度达到≥60 mm（规格为0-100 mm的VAS）判定为咳嗽，并归入术后咳嗽组^[10]^。

### 1.5 LCQ-MC量表信度

本文采用克朗巴赫α系数来验证LCQ-MC量表在肺部手术后患者应用情况的信度^[11]^。评价标准：克朗巴赫α系数小于0.6表示内部一致信度不足，达到0.7-0.8表示量表具有相当的信度，0.8以上说明信度非常好。

### 1.6 咳嗽的诊断标准

最小临床重要差异（minimal clinically important difference, MCID），表示患者觉得有临床意义的最小健康状态变化。其中，慢性咳嗽和急性咳嗽患者在LCQ-MC量表中的最小临床重要差异分别为1.3和2.0^[12, 13]^。

### 1.7 统计学方法

采用Excel 2013软件进行数据管理和SPSS 19.0软件进行统计分析。通过计算克朗巴赫α系数，并作为信度指标来评价量表内多个维度的内部一致性；采用独立样本*t*检验对术后出现咳嗽组与术后未出现咳嗽组的资料进行比较；采用*SNK*（*Student-Newman-Keuls*）法方差分析对不同手术方式之间的资料进行比较。

## 2 结果

### 2.1 肺手术患者入院与术后LCQ-MC分值分析

术前LCQ-MC总分（19.57±1.73）高于术后（17.71±2.72）（*P*=0.041），且差值为（2.34±2.03），大于急性咳嗽与慢性咳嗽的最小临床重要差异（2.0 *vs* 1.3），提示肺手术患者术后具有不同程度咳嗽。生理维度分值在术前（6.32±0.74）显著高于术后（5.39±1.10）（*P*=0.031），然而心理和社会维度分值在手术前后变化不大，无统计学差异，提示患者术后咳嗽的影响主要反应在生理方面，而心理和社会方面受到的影响较小（表 1）。

**1:** 121例肺部疾病患者术前与术后LCQ-MC分值 The mean LCQ-MC score before and after pulmonary operation in patient

	LCQ-MC score			*P*
	Preoperative	Postoperative	Difference*	
Physical	6.32±0.74	5.39±1.10	1.15±0.87	0.031
Psychological	6.47±0.70	6.04±0.99	0.74±0.74	0.073
Social	6.78±0.53	6.29±0.98	0.65±0.82	0.235
Total	19.57±1.73	17.71±2.72	2.34±2.02	0.041
^*^The absolute value of the difference between LCQ-MC scores before and after operation; LCQ-MC: Mandarin Chinese version of the Leicester Cough Questionnaire.				

### 2.2 LCQ-MC量表用于肺手术患者的信度评估

121例患者手术前后的克朗巴赫α系数均大于0.7，提示LCQ-MC量表应用于肺部手术后患者时，其具有良好的信度。经过计算后，手术前后的克朗巴赫α系数与表格创建者Birring等以慢性咳嗽患者为对象所计算的克朗巴赫α系数接近（表 2）。

**2:** 术前及术后克朗巴赫α系数 Cronbach's alpha coefficient in before and after operation

	Birring SS^*^	Preoperative	Postoperative
Physical	0.79	0.72	0.73
Psychological	0.89	0.72	0.79
Social	0.85	0.82	0.78
Total	0.92	0.87	0.89
^*^Birring SS is the creator of Leicester Cough Questionnaire (LCQ).			

### 2.3 术后出现咳嗽与未出现咳嗽患者LCQ-MC分值分析

121例肺部疾病患者术后出现明显咳嗽症状患者为73例（60.33%, 73/121），未出现明显咳嗽症状患者48例（39.67%, 48/121）。咳嗽组患者的术前LCQ-MC分值在（19.31±1.84）显著低于无咳嗽组（19.97±1.46）（*P*=0.038），且主要差异在于生理维度的LCQ-MC分值（*P*=0.015）。术后LCQ-MC值在咳嗽患组（16.67±2.91）显著低于无咳嗽患者（19.30±1.32）（*P*=0.001），而且两组生理维度的LCQ-MC分值也有显著性差异（*P*=0.001）。手术前后LCQ-MC总分的差值两组差值为（2.93±2.25）（大于急性咳嗽与慢性咳嗽的最小临床重要差异2.0 *vs* 1.3），生理维度的分值差值为（1.35±0.95），提示肺术后咳嗽的潜在影响因素可能是手术前患者的咳嗽情况。结果表明，肺部疾病患者手术后出现的咳嗽症状对于患者的健康相关生活质量有影响，主要体现在生理维度，对心理与社会维度的影响较小，比较符合临床实际，所以认为LCQ-MC量表能较好地评价出肺部手术后患者的咳嗽情况（表 3）。

**3:** 肺手术后患者咳嗽状况及LCQ-MC值分析 The mean LCQ-MC score before and after pulmonary operation in patients with postoperative cough group and without postoperative cough group

	LCQ-MC score	Cough (*n*=73)	No cough (*n*=48)	*P*
Preoperative	Physical	6.19±0.80	6.52±0.59	0.015
	Psychological	6.38±0.75	6.61±0.58	0.077
	Social	6.74±0.56	6.84±0.47	0.299
	Total	19.31±1.84	19.97±1.46	0.038
Postoperative	Physical	5.02±1.16	5.94±0.68	0.001
	Psychological	5.66±1.06	6.61±0.49	0.001
	Social	5.99±1.09	6.75±0.54	0.001
	Total	16.67±2.91	19.30±1.32	0.001
Difference	Physical	1.35±0.95	0.86±0.62	0.001
	Psychological	0.91±0.82	0.50±0.53	0.001
	Social	0.90±0.89	0.28±0.55	0.000
	Total	2.93±2.25	1.47±1.22	0.000

### 2.4 肺叶切除患者与非肺叶切除患者的LCQ-MC分值分析

121例肺部疾病手术患者，其中肺叶切除术73例，肺段切除18例，肺楔形切除30例，由于样本量差距较大，故把肺段切除与肺楔形切除患者合并分析，分为肺叶切除术组73例，非肺叶切除术组48例。肺叶切除术组患者术前LCQ-MC分值（19.48±1.63）和非肺叶切除（19.76±1.88）无明显统计学差异（*P*=0.402）; 两组患者术后LCQ-MC值（分别为17.75±2.51，17.79±3.04）无统计学差异（*P*=0.936）；两组手术前后差值两组患者术后LCQ-MC值[分别为（2.27±2.07）、（2.44±1.91）]也无统计学差异（*P*=0.648）。LCQ-MC无法较好区分不同手术方式的患者咳嗽情况，也说明了量表目前在评估咳嗽以外的方面效果较差，需进一步改进（表 4）。

**4:** 肺叶切除和非肺叶切除LCQ-MC值分析 The mean LCQ-MC score of lobectomy and non-lobectomy

	LCQ-MC score	Lobectomy (*n*=73)	Non-lobectomy (*n*=48)	*P*
Preoperative	Physical	6.28±0.72	6.41±0.76	0.336
	Psychological	6.43±0.67	6.56±0.73	0.316
	Social	6.78±0.50	6.78±0.58	0.943
	Total	19.48±1.63	19.76±1.88	0.402
Postoperative	Physical	5.42±1.02	5.36±1.20	0.736
	Psychological	6.06±0.97	6.10±1.00	0.818
	Social	6.26±0.88	6.33±1.15	0.720
	Total	17.75±2.51	17.79±3.04	0.936
Difference	Physical	1.06±0.85	1.28±0.89	0.171
	Psychological	0.73±0.77	0.77±0.70	0.762
	Social	0.73±0.81	0.52±0.84	0.167
	Total	2.27±2.07	2.44±1.97	0.645

## 3 讨论

外科手术后，因麻醉或手术创伤本身，常常导致患者术后不同程度的咳嗽，多数患者术后经过一段时间可以自愈，不需要特殊处理。但胸科术后（尤其是肺部手术后），由于其手术与疾病的特殊性，术后出现咳嗽的程度和持续时间较长，常常会影响到患者出院后的生活质量，有的甚至会发展成顽固性咳嗽。然而，目前临床上关于胸外科术后咳嗽机理及治疗的相关研究较少。因此，能客观有效地评估肺术后患者咳嗽情况，对于咳嗽的治疗已经出院后生活质量的改善有重要临床意义。

目前，主要的咳嗽评估工具主要从咳嗽的严重程度、频率以及健康相关生活质量三个方面评估^[14]^。其中，用于评估咳嗽健康相关生活质量的评估工具主要是LCQ^[11]^和咳嗽生活质量问卷（The Cough-specific Quality life of Life Questionnaire, CQLQ）^[15]^，本文采用的LCQ-MC是中文版的LCQ，该量表由19道题目组成，分为生理、心理和社会三个健康相关维度。LCQ-MC量表已被验证与圣乔治呼吸问卷（St George's Respiratory Questionnaire, SGRQ）以及生活质量测定量表简表（Short Form 36 questionnaire, SF36）有良好的相关性。其操作简易，一般5分钟以内可完成问卷^[16]^，LCQ已被证实能有效评估慢性咳嗽、急性咳嗽、急性上呼吸道感染后咳嗽以及慢性阻塞性肺病（chronic obstructive pulmonary disease, COPD）等^[11, 13, 17, 18]^。LCQ最早是由李斌恺等^[19]^引入中国，用于慢性咳嗽患者的生活质量调查，较好地反应了慢性咳嗽对于患者日常生活、学习工作以及身心健康的影响。如Gao等^[5]^于2014年将LCQ量表翻译成普通话版，即LCQ-MC，并证实LCQ-MC能有效地评估支气管扩张症患者的咳嗽情况。黄佳等^[20]^于2010年首次报道了LCQ-MC量表在右肺癌系统淋巴结清扫术后顽固性咳嗽的应用，LCQ-MC量表在术后1个月的随访中良好地反应了肺癌术后患者的咳嗽情况。

信度主要是评价量表的精确性、稳定性和一致性，即测量过程中随机误差造成的测定值的变异程度大小的一个统计学指标。常用的信度指标有重测信度、分半信度和克朗巴赫α系数。分半信度是指同一量表的调查条目得分一分为二计算的相关系数，不足之处是不同的条目分半而产生不同的计算方法，另一不足是它只适用于条目为二分变量的测验而不适用于项目多重记分的测验。克朗巴赫α系数的性质则相当于是量表所有分半信度的平均值，反映了量表的精确性，也反映了量表各条目间的一致性和稳定性程度，故本研究采用克朗巴赫α系数反应信度。结果显示，术前、术后的各维度与总分的克朗巴赫α系数均大于0.7，且与Birring等^[5, 11]^制定LCQ和GAO YH等翻译LCQ-MC时候计算出的各维度及总分克朗巴赫α系数均相近，说明了LCQ-MC在肺部疾病患者术后咳嗽的情况调查中具有良好的信度，体现了LCQ-MC的稳定性。

LCQ-MC在肺部疾病患者的术后咳嗽情况中，能良好的区分术后的咳嗽情况，术后出现咳嗽的患者组（16.67±2.91）显著低于术后无咳嗽患者（19.30±1.32）（*P*=0.001），进一步分析各维度得分变化，生理维度的分值变化大于心理社会维度的变化，也与我们临床实际相符。同时，通过数据分析还发现术前患者LCQ-MC值在咳嗽组（19.31±1.84）显著低于无咳嗽组（19.97±1.46）（*P*=0.038），且主要差异在于生理维度方面（*P*=0.015），这提示了患者手术前的咳嗽情况很可能是影响术后咳嗽程度的一个潜在影响因素，尚需要进一步的研究来证实。

但是，LCQ-MC在区分不同手术方式后患者咳嗽情况的表现不甚理想，本研究中由于患者三种手术方式样本量差异较大，故把肺楔形切除术与肺段切除术患者合并分析，分析结果显示LCQ-MC无法很好区分肺叶切除术患者与非肺叶切除术患者的咳嗽情况（*P*=0.654）。探究其原因，一方面是样本量仅121例患者，可能是样本量不足导致；另一方面，由于LCQ-MC的条目中无任何手术相关条目，对于不同手术造成的咳嗽区分度较差。

LCQ-MC首次在胸外科肺部疾病手术后患者的咳嗽情况调查中，总体效果是令人满意的，量表操作简单，能明显区分手术后患者的咳嗽情况，但是仍有许多不足之处。首先，由于LCQ主要是为慢性咳嗽患者设计，故很多条目不适用于外科使用，如心理维度条目意思相近，容易造成患者甚至医务工作者的混淆；其次，LCQ-MC不能全方面的反应患者的咳嗽情况，偏重于患者生活质量方面的评估。故在本次调查后，我们将量表进行了改良，但是目前尚在临床数据收集中，尚未经过严格的统计学验证，并打算与其他量表联合应用，使其更加适用于肺部疾病患者。原版LCQ-MC见详附件1（http://www.lungca.org/files/2017-02-OA-0042-supplement1.pdf），改良后量表详见附件2（http://www.lungca.org/files/2017-02-OA-0042-supplement2.pdf）。

综上所述，应用LCQ-MC评估肺部疾病术后患者的咳嗽情况效果良好，但也存在一些不足之处，通过进一步的研究来对量表进行完善、修正，相信改良后的LCQ-MC将能成为一种有效、简洁的肺部疾病术后咳嗽情况的评估工具。
